# Quantum Cascade Laser Absorption Spectroscopy as a Plasma Diagnostic Tool: An Overview

**DOI:** 10.3390/s100706861

**Published:** 2010-07-16

**Authors:** Stefan Welzel, Frank Hempel, Marko Hübner, Norbert Lang, Paul B. Davies, Jürgen Röpcke

**Affiliations:** 1 INP Greifswald, Felix-Hausdorff-Str. 2, 17489 Greifswald, Germany; E-Mails: hempel@inp-greifswald.de (F.H.); huebner@inp-greifswald.de (M.H.); lang@inp-greifswald.de (N.L.); roepcke@inp-greifswald.de (J.R.); 2 Department of Chemistry, University of Cambridge, Lensfield Road, Cambridge CB2 1EW, UK; E-Mail: Daviespbd2@cam.ac.uk; 3 Eindhoven University of Technology, P.O. Box 513, 5600 MB Eindhoven, The Netherlands

**Keywords:** quantum cascade laser, infrared laser, absorption spectroscopy, plasma diagnostics, cavity enhanced spectroscopy, chemical sensing, 07.57.-c, 42.55.Px, 42.62.-b, 52.70.Kz, 82.33.-z

## Abstract

The recent availability of thermoelectrically cooled pulsed and continuous wave quantum and inter-band cascade lasers in the mid-infrared spectral region has led to significant improvements and new developments in chemical sensing techniques using *in-situ* laser absorption spectroscopy for plasma diagnostic purposes. The aim of this article is therefore two-fold: (i) to summarize the challenges which arise in the application of quantum cascade lasers in such environments, and, (ii) to provide an overview of recent spectroscopic results (encompassing cavity enhanced methods) obtained in different kinds of plasma used in both research and industry.

## 1. Introduction

Over the past two decades chemical sensing using laser absorption spectroscopy (LAS) in the molecular fingerprint region from 3 to 20 μm has been established as a powerful *in-situ* diagnostic tool for molecular plasmas [1–6]. The non-invasive, selective and time resolved detection and quantification of transient and stable molecular species provides important information on the gas phase composition and chemistry of complex gas mixtures in electric discharges. However, this diagnostic approach often has seemed more suitable for research laboratories than for industrial process monitoring or control due to the characteristics of the available infrared (IR) light sources. In particular, tuneable, narrow band light sources are desirable for selective high resolution IR-LAS. Apart from line-tuneable gas lasers, parametric frequency conversion (e.g., difference frequency generation or optical parametric oscillators) and semiconductor based lasers have all been successfully employed for spectroscopic studies [7]. In contrast to non-linear frequency conversion, which typically covers only the 3 to 5 μm range and requires optical pumping and thus an additional (near-IR) light source [8], direct stimulated emission from 3 to 30 μm is achieved across the band gap in tuneable diode lasers (TDLs) composed of lead salts. Although the design and hence the performance of this class of multi-mode IR lasers has been improved and may now provide output powers of a few milliwatts cryogenic cooling is still essential and remains a significant limiting factor for wider application [9].

In the 1990s laser action based on transitions between confined quantum states in cascaded inter-subband, inter-miniband or inter-band structures, was demonstrated [10–12]. Combined with integrated distributed feedback (DFB) gratings the emission wavelength of this new class of thermoelectrically (TE) cooled unipolar semiconductor lasers, often called quantum cascade lasers (QCLs), can be custom-tailored from the mid-IR to the terahertz range. A lower wavelength limit of 3.4 μm has been reported for QCLs [13], being determined by the band gap discontinuity of the III-V materials. Consequently, the interesting C-H stretching region around 3 μm requires inter-band cascade lasers (ICLs) or the established mid-IR sources mentioned above to be applied. Initially, near room temperature operation of QCLs was limited to pulsed mode operation with low duty-cycles of a few percent. Continuous improvement, specifically of the active region design, and heat management, has made continuous wave (cw) operation possible [14–16], which has been reviewed elsewhere [17–22]. Recent TE cooled devices provide from hundreds of milliwatt up to watts of cw radiation power. DFB-QCLs are capable of continuous mode-hop free wavelength tuning. On the other hand their total emission range is typically limited to less than 7 cm^−1^ (between ±30 °C) compared with an (incomplete) coverage of hundreds of wavenumbers in the case of temperature tuned multi-mode lead salt TDLs. Hence the use of QCLs requires a relatively precise selection of the laser.

Meanwhile the availability of QCLs and ICLs as substitutes for lead salt TDLs has led to a rapid development of IR-LAS from a niche position to a standard diagnostic technique. In particular field applications of trace gas sensors are of increasing interest, e.g., for isotope measurements [23], atmospheric sensing [24], explosives detection [25] or breath analysis [26]. Additionally, the spectral characteristics of QCLs facilitated progress in non-spectroscopic applications such as frequency metrology [27], free-space optical communication [28] or near field microscopy [29]. In contrast QCL absorption spectroscopy (QCLAS) has only recently been recognized as an effective plasma diagnostic tool [30,31]. Further development of sophisticated plasma process monitoring [32] and control [33] devices in industrial environments should be forthcoming due to the room temperature operating capabilities of such spectrometers.

Several approaches have been taken to increase sensitivity, such as multiple pass cells [*i.e.*, for direct absorption spectroscopy (D-AS)], modulation schemes, encompassing wavelength (WM) or frequency modulation (FM), or high finesse optical cavities [34]. Techniques based on resonant optical cavities may be categorized and distinguished by their detection principle: In cavity ring-down spectroscopy (CRDS) the decay of light leaking out of a cavity is monitored in time [35]. In contrast, in cavity enhanced absorption spectroscopy (CEAS) [36]–sometimes also called integrated cavity output spectroscopy (ICOS) [37]–the steady-state transmission or integrated transmitted intensity through the cavity is observed as a function of the laser frequency. Cavity excitation is achieved by pulsed or cw light sources. The basic detection schemes might be combined with WM or FM. The latter has led to a substantially improved sensitivity which is known as noise-immune cavity enhanced optical heterodyne molecular spectroscopy (NICE-OHMS) [38]. A detailed discussion of specific forms of optical cavity based methods is beyond the scope of this review. A comprehensive introduction has recently provided by Berden and Engeln [39]. A brief summary focusing on the combination of cavity enhanced techniques with QCLs may be found in [40].

The sensitivity of an experiment is typically assessed by the signal-to-noise ratio (*SNR*). Transforming an absorption signal *I*_abs_ into the transmitted intensity, *I*, through the sample and assuming weak absorption for Beer-Lambert law, *I* ∼ exp(−*kL*_eff_) ≈ 1 − *kL*_eff_, where *k* is the absorption coefficient and *L*_eff_ an effective absorption length, yields an estimate of the *SNR*:

(1) $$\mathrm{SNR}=\frac{I_{\text{abs}}}{\Delta I}=\frac{1-I}{\Delta I}\approx \frac{I_{0}}{\Delta I}{\mathrm{kL}}_{\text{eff}}.$$

Provided that a minimum *SNR* = 1 is achieved with a noise level of Δ*I*, an expression for the minimum detectable absorption coefficient *k*_min_ can be deduced from (1):

(2) $$k_{\min}=\Delta k\approx \frac{\Delta I}{I_{0}L_{e\text{ff}}}.$$

**Figure 1.**
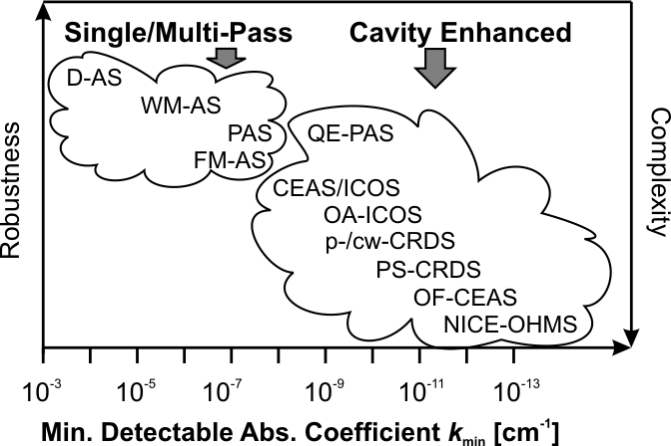
Comparison of selected absorption spectroscopy (AS) techniques according to their typically achieved minimum detectable absorption coefficients, *k*_min_, their robustness and complexity. Abbreviations which are not explained in the text can be found in appendix A.1 (adapted from [42]).

Figure 1 illustrates typical values of *k*_min_ which can be achieved with direct or with modulation techniques, respectively, and involves single or multiple pass configurations as well as methods based on optical cavities. In general, another quantity, namely the noise equivalent absorption, *NEA*, is used to estimate the detection limit:

(3) $$\mathrm{NEA}∼\Delta {\mathrm{kf}}^{-1/2},$$

which also takes the repetition rate of the measurements *f*, *i.e.,* the number of averages into account. It should be noted that the *NEA* is not defined consistently throughout the literature. The expressions usually employed are discussed in [41]. The *NEA* defined in (3) and provided for the CEAS experiments would correspond to the “per scan” category in [41].

The present paper is intended to give an overview of recent achievements in mid-IR chemical sensing based on the application of QCLAS techniques. The focus here is on plasma environments. This article is divided into two main parts: Section 2 concerns the pulsed mode of operation (p-QCLAS) and summarizes the challenges which are connected with using this kind of laser, particularly in low pressure plasmas. The time resolution achievable is discussed and special attention is paid to industrial applications. Section 3 highlights some results obtained with cw lasers (cw-QCLAS) encompassing cavity enhanced methods. A summary and future prospects are presented in Section 4.

## 2. Pulsed QCLs

### 2.1. General Spectroscopic Issues

Early experiments with (pulsed) QCLs combined short laser pulses of the order of a few nanoseconds with the conventional method of scanning TDLs by ramping a DC current [43], which is often referred to as *inter* or short pulse mode. Strictly speaking spectral tuning is accomplished by temperature induced changes in the refractive index of the laser which tunes both the spectral gain and to a lesser extent the period of the DFB grating [18]. Impressing a sub-threshold current ramp is therefore an indirect method of tuning the QCL. In contrast to lead salt lasers QCLs require up to an order of magnitude higher compliance voltages, leading to a considerably increased input power fed to the device. This in turn makes the device temperature, and thus the refractive index, vary, particularly during a laser pulse. Consequently, an inherent frequency-down chirp is observed for pulsed QCLs. This was later exploited by using single long laser pulses (*i.e.*, some hundred nanoseconds) to acquire entire absorption spectra which is also known as the *intra* pulse mode [44,45].

Apart from pulse-to-pulse intensity fluctuations, a phenomenon well-known from other pulsed radiation sources, increased effective laser line widths of up to 1.2 GHz (0.04 cm^−1^, full width at half maximum) were reported as a major drawback of p–QCLAS [46]. McCulloch *et al.* estimated the effective QCL spectral width, and hence the spectral resolution Δν, of a pulsed spectrometer to be ∼ (*C* · *α*)^1/2^, where *α* is the spectral sweep rate (d*f*/d*t*) and *C* a current pulse shape-dependent constant [47]. Assuming a rectangular and a Gaussian time-window *C* equals 0.886 and 0.441, respectively. Hence, *C* = 1 may be considered as an upper limit in the following discussion. It should be noted that McCulloch's estimate is true for an optimized pulse width Δ*t* = Δ*t*_opt_ (best aperture time). In general, the spectral width Δν is governed either by the uncertainty relation, Δν · Δ*t* ≥ *C*, or by the frequency chirp of the laser, Δν = *α* · Δ*t*. For extremely short pulses (Δ*t* = Δ*t*_short_ < 5 ns) the spectral band width is Fourier transform limited Δν_short_ ∼ *C*/Δ*t*_short_. For longer pulses Δ*t* = Δ*t*_long_ the frequency chirp of the laser sets the fundamental limit Δν_long_ = α · Δ*t*_long_ and the spectral width clearly exceeds the theoretical value given by the uncertainty relation (*i.e.,* Δν_long_ ≫ *C*/Δ*t*_long_). McCulloch suggest therefore the best aperture time Δ*t*_opt_ for which the Fourier transform limited band width equals the frequency chirp (*i.e.*, Δν_short_ = Δν_long_) in order to estimate the spectral resolution of pulsed spectrometers. In practice, the pulse widths Δ*t* = *t*_on_ which are available from QCL drivers are usually higher than this best pulse width (*t*_on_ > Δ*t*_opt_). Hence, (*C* · *α*)^1/2^ provides a lower limit approximation of the effective line width, since Δν often remains chirp limited (∼ *α* · *t*_on_).

The increased laser line widths are accompanied by a relatively fast chirp α (about tens of MHz per ns). The fast chirp rate combined with the high laser output power enables non-linear absorption phenomena to be observed. In other words, linear absorption spectroscopy governed by the Beer-Lambert law may be invalid. Furthermore, the combination of pulsed QCLs with highly sensitive cavity ring-down experiments is typically hampered due to an insufficient intensity build-up inside the high finesse cavity and multi-exponential ring-down transients [40].

**Figure 2.**
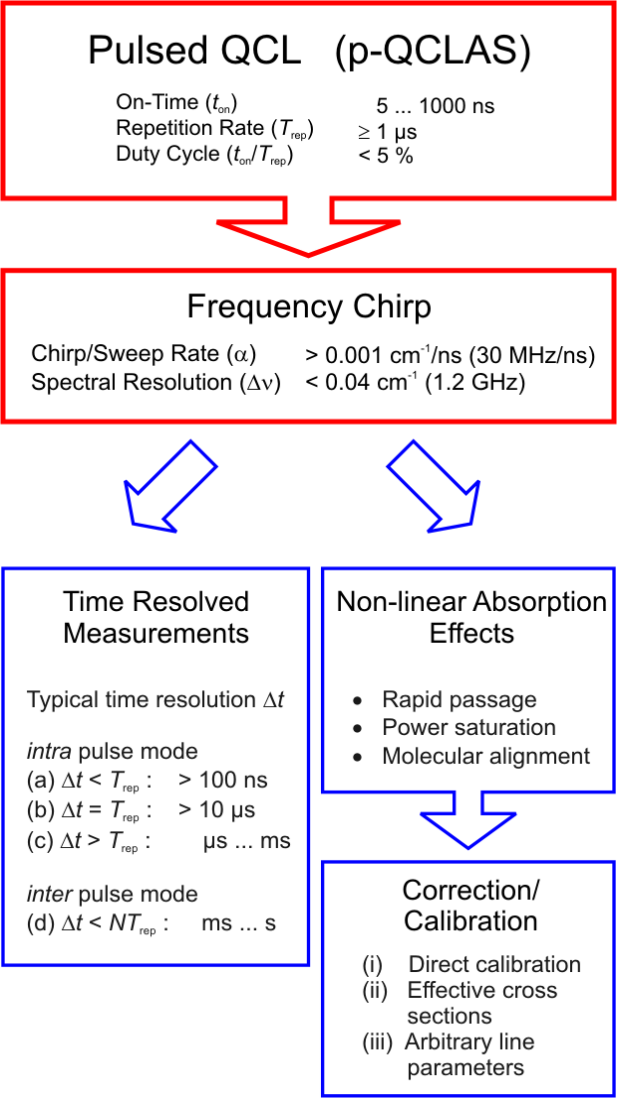
Overview of issues occurring in p–QCLAS.

Nevertheless, the frequency chirp also offers advantages for chemical sensing: entire absorption spectra of up to ∼ 1 cm^−1^ (30 GHz) are recorded during a few hundred nanoseconds pulse width which fits very well to highly time resolved measurements of rapidly changing chemical processes. Studies under turbulent gas phase conditions are facilitated, because the data acquisition time is shorter than random fluctuations in the sub-millisecond range. Figure 2 collects the main issues and typical figures of merit that are connected with the application of pulsed QCLs. These are detailed in the following sections: Potential non-linear absorption effects and their treatment are briefly discussed in Sections 2.2 and 2.3 Time resolution issues are concerned in Section 2.4 Recent examples of these aspects of p-QCLAS will be presented (Section 2.5).

### 2.2. Non-Linear Absorption Phenomena

Pulsed QCLs combine both high (peak) output power of linearly polarized IR radiation and rapidly swept emission frequencies. In particular under low pressure conditions when there are reduced relaxation rates, *γ*, of ro-vibrational molecular levels, these two laser properties may lead to non-linear phenomena, e.g., power saturation and rapid passage effects, respectively.

The sweep rates (*α*) measured in conjunction with pulsed QCLs are sufficiently high (≥ 0.001 cm^−1^/ns or 30 MHz/ns) to observe rapid passage effects for optical transitions under low pressure conditions [48,49]. Rapid passage is generally accomplished by sweeping the source or detuning the resonance frequency by means of external fields on time scales shorter than 1/γ and was, for example, described for magnetic resonance transitions when the external field is rapidly swept over the transition [50]. Assuming pressures below 10 mbar yields typical collisional half widths of < 1 × 10^−3^ cm^−1^ which corresponds to relaxation rates γ below 30 MHz. Characteristic Doppler broadening in the mid-IR is approximately of the same order (1 × 10^−3^ cm^−1^). Thus, it can be concluded from the above mentioned laser sweep rates of ∼0.001 cm^−1^/ns that the QCL emission stays about a nanosecond in resonance with an absorption feature [51]. Such a 1 ns interaction time is clearly shorter than 1/γ (> 33 ns in this example) which is the important criterion for the appearance of rapid passage effects. The normalized sweep rate, *A* = *α/γ*^2^, which is often used to characterize such processes, would be 30. Hence the criterion for rapid passage phenomena, *A* ≫ 1 [50], is fulfilled. Since the lower limit chirp rate was assumed here this suggests that rapid passage obstacles are present with all pulsed QCLs at low pressures.

If the laser intensity and thus the electric field amplitude *E*_0_ is sufficiently high, power saturation, described by the saturation parameter, *Σ* = [*μE*_0_/(h*γ*)]^2^ [52], where h is Planck's constant, may be observed, *i.e.*, a substantial population transfer occurs between the lower and the upper level of the transition with the dipole moment *μ*. For strong absorption features (line strength *S* > 10^−20^ cm/molecule for most abundant isotopes) *Σ* may approach unity at low pressures. This requires only about 500 mW peak power—which is equivalent to a few mW average power if a duty-cycle in the percent range is applied to the QCL—for a collimated laser beam of a few millimeters in diameter. The combination of strong power saturation with the rapid passage effect is often referred to as the adiabatic rapid passage effect. The corresponding figure of merit is *A/Σ* [48,49] and will be ≪ 1 which is equivalent to an increased interaction time of the chirped laser with the transition, *i.e.,* strong optical pumping occurs. As long as *A/Σ* ≫ 1, also known as the linear rapid passage regime, the interaction time is relatively short. Typical frequency-down chirped QCL pulses are within the linear regime which follows from the estimates for *A* and *Σ* and has recently been confirmed [53].

It should be noted that rapid passage and power saturation are separate non-linear phenomena. The previous discussion is summarized in this respect in Table 1. While rapid passage phenomena are present for almost all pulsed QCLs at low pressure, power saturation might be absent. In practice the discrimination between both effects is relatively difficult, because both simultaneously disturb the line shape and the integrated absorption coefficient. Moreover, absorption features may be convolved with the limited analogue bandwidth of the detection system, since the typical sweep rates require high bandwidth instrumentation in the GHz range which are now becoming available for the mid-IR by means of TE cooled HgCdTe detectors.

A theoretical description based on optical Bloch equations with special attention paid to inhomogeneous (Doppler) broadening, which is negligible in magnetic resonance experiments, has been proposed [48,51,54]. Molecular alignment caused by the linearly polarized laser beam has to be considered in the calculations [48]. However, it was omitted here since the focus of this review is slightly different, namely on obtaining (correct) molecular concentrations from *in-situ* measurements without post-processing. These calculations are not useful for retrieving corrected number densities in real-time from disturbed absorption features. A detailed treatment of the theory is thus beyond the scope of this paper, particularly due to the fact, that it does not facilitate a straightforward correction of underestimated number densities in multi-species environments such as plasmas. Therefore, in what follows, more practical approaches are summarized.

### 2.3. Quantification of Number Densities

The conclusion from the previous section is that integrated absorption coefficients obtained with pulsed QCLs under low pressure conditions usually require a correction since non-linear absorption phenomena are present. Notably, the ratio of the integrated absorption coefficient of two transitions is not affected which allows a correct determination of gas temperatures with p-QCLAS [55]. Straightforward approaches to determining the absolute ground state densities of molecular species in plasmas may be characterized as:

- calibration of individual line strengths *S* or absorption coefficients σ(Section 2.3.1.),
- employing an effective absorption coefficient σ_eff_ determined for a specific spectral micro-window <ν> (Section 2.3.2.), or
- using optimized arbitrary line positions and strengths for complex spectra (Section 2.3.3.).

Basically, all the methods (i)—(iii) are based on calibration of conditions, e.g., pressure, temperature, absorption path length, close to the operating parameters.

#### 2.3.1. Calibration of individual lines

**Figure 3.**
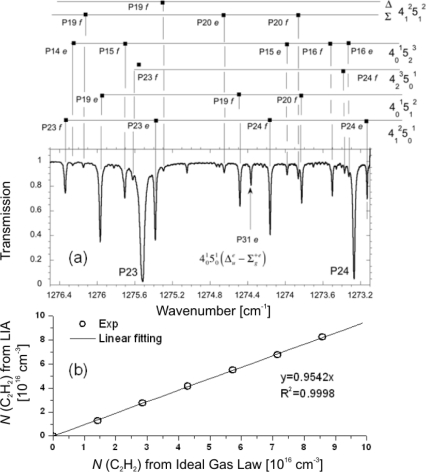
Sample spectrum and calibration curve of a stable molecular species, C_2_H_2_, obtained with a pulsed QCL at elevated pressure. **(a)** Transmission spectrum of a room temperature sample (19 cm absorption path) exhibiting no rapid passage features due to higher pressure (>50 mbar [30]). **(b)** C_2_H_2_ number densities determined from the line integrated absorption coefficient (LIA) of line *P*(23,*e*) for various experimental conditions at 298 K [56] (reprinted with permission from [56]. Copyright 2009, American Institute of Physics).

**Figure 4.**
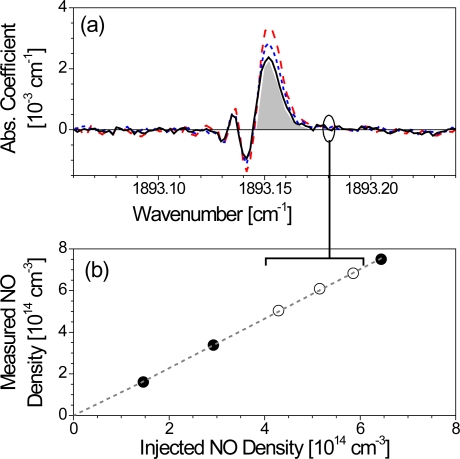
Sample spectrum and calibration curve of a stable molecular species, NO, obtained with a pulsed QCL under low pressure conditions. **(a)** Perturbed absorption coefficients of NO measured at 2.66 mbar (50 cm absorption length) for different mixing ratios (dashed—1.00%, dotted —0.80%, solid—0.67%. The patterned area was used to calculate number densities (at 296 K) which are plotted in **(b)** as open symbols (full symbols represent values at different pressures). The resulting correlation was then used to correct for non-linear absorption phenomena (adapted from [31]).

Figures 3 and 4 show two examples for calibrating (unblended) absorption features of known line strength (case i). Figures 3a and b illustrate experiments with acetylene (C_2_H_2_) at elevated pressures [56] while a different situation is depicted in Figures 4a and b showing absorption spectra of nitric oxide (NO) at low pressure [31]. Typical rapid passage undulations normally present on the low frequency side of absorption lines are absent in the C_2_H_2_ spectrum (Figure 3a), because the collisional relaxation rates are sufficiently high under these conditions (>50 mbar) to lower the normalized sweep rate *A*. Power saturation should also be negligible since the gradient of the correlation between measured and calculated integrated absorption coefficients is close to 1 (Figure 3b).

In Figure 4a the rapid passage oscillations are clearly visible. Absolute number densities were obtained by integrating the positive part of the absorption coefficient and subsequently corrected by a factor deduced from a plot of the measured against the injected concentration (Figure 4b). The linear correlation in Figure 4b, which is valid over about one order of magnitude, reveals that power saturation can also be neglected. If present, this effect would usually be identifiable from a non-linear and reduced gradient [54].

Calibration method (i) is ideally performed for unblended lines and requires line strength data for all absorption features implying that the gas temperature is known. These *S*(*T*) values are individually corrected by a factor which includes all deviations from the linear Beer-Lambert law. In special cases where no line strength data or absorption cross sections are known but molecular constants for the species of interest are available, a spectral simulation can be applied to retrieve *S* as demonstrated by Hancock *et al.* for the CF_3_ [57]. This approach is particularly useful for transient molecules. While reference gas mixtures of stable species can easily be provided for calibration experiments, pre-defined concentrations of transient species might be difficult to achieve. In these cases a careful simulation is a promising option.

#### 2.3.2. Effective absorption cross sections

**Figure 5.**
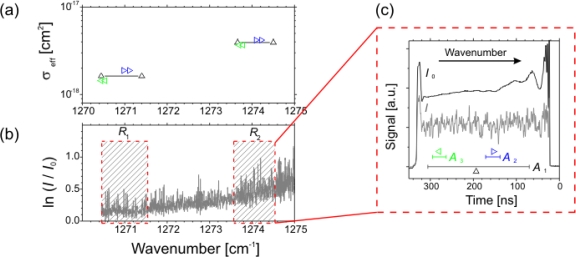
**(a)** Example of retrieving effective absorption cross sections from two different spectral ranges within the ν_3_ band of CF_4_. **(b)** Tuning the QCL heat sink temperature stepwise yielded a total spectral sweep of about 6 cm^−1^. **(c)** Single baseline (*I*_0_) and transmission spectra (*I*) of CF_4_ (0.1 mbar, 90 cm absorption path) obtained by applying long QCL pulses of 300 ns. Effective absorption cross sections σ_eff_ were measured in two spectral ranges (*R*_1_ and *R*_2_). Calibration results from 3 different spectral micro-windows (*A*_1_—triangle up, *A*_2_—triangle right, and *A*_3_—triangle left) within a specific range *R* were in good agreement **(a)** (adapted from [58])

For the majority of relevant polyatomic molecules neither high resolution absorption cross sections nor spectral data for additional calculations are reported in the literature. The absorption spectra are commonly complex in nature and as a result, they lack any appearance of the rapid passage phenomena (see Figure 5 or [32,57,58]). It has been demonstrated that defining effective absorption cross sections σ_eff_ within a spectral micro-window <ν> in both the *inter* or *intra* pulse method may also lead to an adequate calibration (case ii) [32,58]. Absolute values for σ_eff_ are obtained from a measurement of the incident and transmitted intensity, *I*_0_ and *I*, of a standardized gas sample of known number density *n* and absorption length *L*:

(4) $${n\sigma }_{\mathrm{eff}}(\langle \nu \rangle )=\frac{1}{L}\ln\left(\frac{I_{0}(\langle \nu \rangle )}{I(\langle \nu \rangle )}\right).$$

Figure 5b shows a CF_4_ overview spectrum merged from several chirped QCL pulses. For two of these *intra* pulse sweeps (*R*_1_ and *R*_2_) spectral micro-windows were defined, namely *A*_1_, *A*_2_ and *A*_3_ (Figure 5c), for which effective cross sections could be determined. Although the spectral coverage of the micro-windows is different the σ_eff_ <ν> values are in good agreement (Figure 5a) [58].

Note that σ_eff_ may have a strong temperature dependence (CF_4_, C_3_F_8_ [58]), whereas this is absent for other molecules (SiF_4_ [32]). Calibration is therefore preferably carried out close to the experimental conditions to avoid inaccuracies due to line broadening and non-linear absorption effects, since σ_eff_ factors in such phenomena. Hence, the effective cross sections are specific for the measurement system used. The advantage of this approach for further data analysis is that absolute number densities can be inferred just from the absorption coefficient (right hand side of Equation 4) without considering and integrating over all individual unresolved absorption lines in the complex spectrum. This reduces the computation effort, which is an important consideration during time resolved measurements.

#### 2.3.3. Arbitrary line parameters

**Figure 6.**
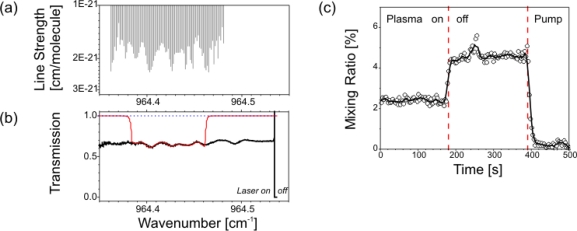
**(a)** Calibrated BCl_3_ stick spectrum. **(b)** The line strength data from (a) were used to achieve a fit (thin solid line) to the measured transmission spectra (heavy solid line) through a MW reactor. The laser signal was turned off momentarily to record the detector offset signal. All *inter* pulse spectra were normalized to a previously acquired baseline (dotted line). **(c)** Time resolved BCl_3_ mixing ratios measured in a H_2_/Ar/BCl_3_ MW discharge (2 mbar) using the advanced fit procedure for complex spectra depicted in **(b)** (adapted and enlarged from [63]).

Another, more advanced method (iii) of processing densely packed calibration spectra was proposed by Harward *et al.* [59–61]. After estimating line strengths for an arbitrarily chosen number of lines a blended line absorbance spectrum is calculated and compared with the calibration spectrum. The strengths are adjusted until a best match is achieved. It is in fact a hybrid approach based on the advantages of methods (i) and (ii): conventional molecular line parameters are employed as in case (i). However, this approach does not concern the exact set of unresolved lines. The number of individual lines used is minimized to reduce the computation effort (*cf*. case ii). Additionally, the obtained solution for line positions and strengths is not unique. Figure 6a and b illustrate the procedure for a BCl_3_ spectrum obtained with an *inter* pulse QCLAS spectrometer. A reasonable fit to the measured data was achieved with the stick spectrum shown in Figure 6a. If a list of molecular line parameters is established, it can be implemented straightforwardly using the *TDL Wintel* software package providing *inter* pulse laser tuning, data acquisition and real-time analysis [62]. The time trace of the BCl_3_ concentration in a low pressure microwave (MW) discharge (Figure 6c) was recorded in this way.

### 2.4. Time Resolution in p—QCLAS

**Figure 7.**
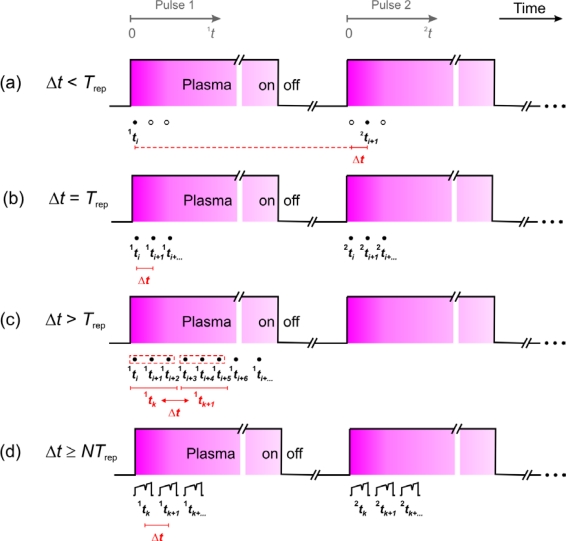
Schematic diagrams of common time resolved p-QCLAS approaches for repetitive (plasma) processes. Circles symbolize single chirped laser pulses, *i.e.*, entire spectra; full circles represent recorded spectra. The necessary QCL repetition period is *T*_rep_. **(a)** Ultimate time resolution Δ*t* down to the QCL pulse width, *t*_on_, may be achieved with delayed trigger experiments; Δ*t* follows from the difference between the delay steps (symbolized by full and open circles). **(b)** Recording each laser pulse yields straightforwardly a time resolution of *T*_rep_. **(c)** Averaging of successive QCL spectra during a plasma pulse (binning) yields effective time steps *^m^t_k_*. Their difference determines Δ*t* and is in fact the number of binned spectra (here: 3) multiplied by *T*_rep_. **(d)** *Inter* pulse spectra (symbolized by several miniature spectra) consist of *N* short pulses. Each entire sweep is described by effective time steps *^m^t_k_* relative to the plasma pulse. The lower limit time resolution is *N · T*_rep_, *i.e.,* the essential time for recording one *inter* pulse sweep.

The achievable time resolution Δ*t* of QCL spectrometers is an important aspect in plasma diagnostics, particularly for studying kinetic phenomena. The fundamental limit is theoretically set by the pulse width *t*_on_ of the QCL which is often in the sub-microsecond range. For this reason, QCLs are considered to be superior to lead salt lasers for which the typical current ramp tuning enables a millisecond time resolution to be achieved. Employing the *intra* pulse method an entire spectrum is acquired within the laser pulse width *t*_on_. However, the reasonable time resolution in p-QCLAS is also affected by other parameters. Particularly, the pulse repetition rate and the *SNR* have to be considered. The upper limit of the repetition rate is thereby strongly dependent on the pulse width *t*_on_, since a maximum duty cycle of a few percent has to be imposed to prevent the QCL from thermal damage. For a QCL pulse width of 100 ns the repetition period should usually be of the order of tens of microseconds. It is therefore clear that the repetition period *T*_rep_ of laser pulses is the crucial parameter and determines the data acquisition approach for time resolved studies as well as the required instrumentation. Four typical cases a–d can be distinguished and are pictured in Figure 7 for a repetitive process, e.g., a pulsed discharge. The following discussion is based on a sequence of plasma pulses which are described by their characteristic time scale, *^m^t*, where *m* = 1, 2 denotes the number of the plasma pulse.

If a time resolution below the laser repetition period is required (Δ*t* < *T*_rep_, Figure 7a), a delayed trigger scheme has to be applied [31,57]. By changing the delay time after igniting different plasma pulses the discharge is probed with Δ*t* = ^2^*t*_*i*+1_ – ^1^*t_i_* and thus Δ*t* is set by the difference in delay times for different plasma pulses *m*. The lower limit is, of course, given by the laser pulse width (Δ*t* ≥ *t*_on_). The *SNR* may be increased by averaging several single shot spectra of the same delay time (<^1^*t_i_*, ^2^*t_i_*, ^3^*t_i_*, …>).

A more straightforward implementation of inherently lower time resolution is shown in Figure 7b, where Δ*t* equals the pulse repetition period *T*_rep_ and may yield a Δ*t* of about 100 μs up to the millisecond range [58]. In this way, an entire discharge pulse can be studied at once with Δ*t* = ^1^*t_i_*_+1_ – ^1^*t_i_* = *T*_rep_ which is also the main advantage over highly time resolved measurements (a). Similar to (a), a better *SNR* is achieved by spectral averaging of corresponding single shot spectra of the same time step from successive plasma pulses *m* (<^1^*t_i_*, ^2^*t_i_*, ^3^*t_i_*, ...>).

Other data acquisition approaches (cases (c) or (d) in Figure 7) should be employed, if an even lower time resolution (e.g., Δ*t* > 100 ms) is required. Laser repetition periods longer than tens of milliseconds may cause additional intensity noise introduced by the controller of the TE cooler which typically have response times of the same order. Therefore *T*_rep_ is often kept in the 100 μs range and a number of successive QCL *intra* pulse spectra, recorded with *T*_rep_, are averaged [56]. In this case, several signals from ^1^*t_i_*, ^1^*t_i_*_+1_, ^1^*t_i_*_+2_ are binned to obtain a higher *SNR* at effective time steps ^1^*t_k_*, ^1^*t_k_*_+1_, (Figure 7c). The achievable time resolution is consequently dependent on the number of spectra (*p*) considered for binning Δ*t* = ^1^*t_k+1_* – ^1^*t_k_* = *pT*_rep_ (*cf*. Figure 7c).

All methods (a–c) discussed above are based on long QCL pulses (*intra* pulse mode). Applying the short pulse mode of operation also enables a reduced repetition period of the laser pulses, *T*_rep,_ of a few microseconds to be used. A spectral sweep, *i.e.*, an entire spectrum, comprises *N* individual QCL pulses (spectral data points). A reasonable spectral sweep in combination with an adequate resolution requires several hundred pulses (*N* ≥ 100). The necessary time for recording a single sweep is ^1^*t_k_* = *N · T*_rep_, where ^1^*t_k_* is again an effective time step representing the entire spectral sweep. The time resolution can thus not be better than the acquisition time for a full sweep Δ*t* = ^1^*t_k+1_* – ^1^*t_k_* = *N · T*_rep_ (Figure 7d). It is often in the millisecond range and usually does not fall below case (b), although the *intra* pulse method requires considerably increased *T*_rep_

To summarize, the only solution for obtaining a reasonable time resolution below ∼200 μs is the delayed trigger method (a), while other approaches are limited either by the repetition period of the QCL (b) or the number of QCL pulses, *p* or *N*, which are essential to obtain an entire spectrum ((c) and (d)). Due to the inherent pulse-to-pulse intensity fluctuations of QCLs spectral averaging is desirable. An increase in the *SNR* is provided by binning of several successively acquired *intra* pulse spectra (c) [30]. The effective time resolution is lowered to the millisecond range making this method appropriate for relatively slow processes. The *inter* pulse mode (d) is also commonly combined with averaging of successive spectral sweeps. Provided a repetition period of *T*_rep_ = 2 μs is used in an experiment using the *inter* pulse method where the sweep consists of 500 spectral data points, *i.e.,* QCL pulses, yields an acquisition time of 1 ms for a single spectrum. Assuming further that 1,000 individual spectra are averaged leads to a time resolution of ∼1 s [32,64].

**Figure 8.**
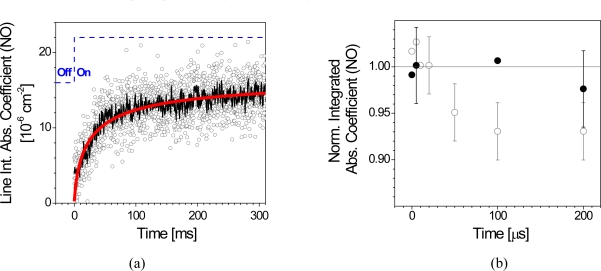
**(a)** Line integrated absorption coefficient (LIA divided by *L*) of NO during the first 300 ms of a pulsed RF plasma. Measurements were performed every 0.2 ms (open circles). Binning of 6 spectra (thin solid line) yields a better *SNR* and an effective 1 ms time resolution. The trend (heavy solid line) serves as a guide to the eye. **(b)** Normalized LIA of NO measured during the first 200 μs of a pulsed 1 ms DC discharge. The uncertainty of single shot results (full black circles) could be reduced by averaging over 5 plasma cycles (open grey circles) at the expense of a drastically increased measurement and post-processing time (enlarged from [31]).

Figure 8a illustrates an example of spectral binning applied to a slow plasma-surface-interaction process: the line integrated absorption coefficient (LIA) of NO:

(5) $$\text{LIA}=\mathrm{nSL}\;=\int_{\mathrm{line}}\ln(\frac{I_{0}(\langle \nu \rangle )}{I(\langle \nu \rangle )})d\nu$$

divided by the absorption length *L* was measured with a QCL repetition period of 0.2 ms in a pulsed 2 s radio frequency (RF) plasma especially designed to study slow surface contributions [65]. Combining 6 spectra already reduces the scatter by a factor of 4 without loosing essential information on the time dependence. In this case the trend with an effective Δ*t* of 1 ms is still derived from only one plasma pulse which is an advantage over all other suggested acquisition schemes. These require several, and by implication, stable discharge events and thorough post-processing of the acquired data to obtain results with an increased *SNR* [58].

The stability of the process is of special importance for highly time resolved measurements, since usually only one spectrum is acquired per plasma pulse (*cf*. Figure 7a). Additional averaging therefore drastically increases the total measurement time. Figure 8b shows the normalized LIA of NO during the early phase of a 1 ms DC plasma which was investigated with Δ*t* down to 5 μs. While under flowing gas conditions each data point was calculated from a spectrum averaged over 5 discharge pulses, reducing the relative error by using static conditions (closed valve) was not feasible due to the lower stability of the discharge [31].

### 2.5. Plasma Diagnostics Using p—QCLAS

#### 2.5.1. Evolution of the gas temperature in a pulsed plasma

A pulsed Ar DC plasma containing ∼ 1 % NO was studied with a time resolution of Δ*t* ≥ 1 μs requiring the delayed trigger method to be applied. Measurements were performed at 1,897 cm^−1^ (5.27 μm) in direct single pass absorption spectroscopy along the 50 cm long symmetry axis of a cylindrical discharge tube. A detection limit of the order of 5 × 10^13^ cm^−3^ was achieved by averaging 5 individual spectra. Since non-linear absorption phenomena were present alongside the low pressure conditions (2.66 mbar) a calibration of the NO line strength data was performed at 296 K.

Since the line strength is temperature dependent, *S*(*T*), the commonly elevated gas temperatures in plasmas may cause a significant deviation from the calibrated value *S*(296 K). On the other hand plasma diagnostic studies commonly lack knowledge of the gas temperatures which in turn hampers an adequate correction and increases the uncertainty in retrieved number densities. The experimental arrangement in this study enabled measurements to be carried out under flowing and static gas conditions (*i.e.*, open and closed valves) at approximately constant pressure *p*. During the plasma pulse the gas is heated up to the same value in static and flowing conditions. According to the ideal gas law *n* ∼ *p*/*T* for a nearly unchanged pressure the number density of the neutrals including NO is depleted supported by the constant gas flow through the tube whereas this is circumvented when the valves are closed. Comparing the absorption signals of both static and flowing conditions therefore provides a means for estimating increase in the gas temperature.

A systematic difference in the LIA normalized to room temperature (*i.e.*, plasma off) conditions was found between both gas regimes (Figure 8b). A temperature increase of ∼40 K with a characteristic heating time of the same order as the 1 ms plasma pulse was estimated and could be confirmed with a complementary model calculation. The model could then be used to determine *T*(*t*) and consequently *S*(*T*(*t*)). The line strength was found to be considerably affected, *i.e.*, lower by ∼15 %, though the temperature increase was relatively moderate [31].

Firstly, this underlines the relevance of even small temperature effects in plasma diagnostic studies, because the main reason for the observed decrease in the normalized LIA during the early plasma phase was found to be the drop in *S*(*T*). Secondly, this kind of p-QCLAS experiments using a carefully selected transition and its temperature dependence accompanied by a complementary method to establish the number density (either experimentally or theoretically) may serve as a highly time resolved, non-invasive and sensitive temperature probe for plasma diagnostic purposes.

#### 2.5.2. Detection and quantification of hydrocarbons in diamond deposition processes

For the majority of plasma processes a straightforward estimate of the gas temperature as proposed in the previous section cannot be achieved. Static gas conditions or a constant pressure may not be available. Additionally, strong inhomogeneities, e.g., in densities or the gas temperature, are often present. For such discharge conditions chemical modeling is required. The calculations may also involve a temperature profile which is used to determine *S*(*T*). Further analysis of the LIAs yields then molecular number densities.

The C_2_H_2_ ⇔ CH_4_ inter-conversion in plasma enhanced chemical vapor deposition (PE-CVD) of diamond has recently been studied in detail [30,56] and is an example for the above mentioned issues. The approach was two-fold namely experimental and theoretical in nature. Direct absorption spectroscopy in single pass configuration using a pulsed QCL in the *intra* pulse mode was applied. A 2 μs laser pulse provided a mode-hop free spectral sweep of ∼4 cm^−1^ centered around 1,275 cm^−1^ (7.84 μm) and thus ground state and vibrationally excited levels of both main stable products CH_4_ and C_2_H_2_ could be observed. A 2D model was established and refined revealing gas temperatures clearly higher than 2,000 K in the plasma core and close to room temperature in the reactor periphery where *T ≈* 330 K.

The conversion of C_x_H_y_ precursors, such as CH_4_, C_2_H_2_ as well as higher hydrocarbons (x > 2), in C_x_H_y_-(7%) Ar-(balanced) H_2_ gas mixtures into CH_4_ and C_2_H_2_ was measured for several process conditions and with millimeter spatial resolution. Quantification of the measured hydrocarbons in the early article was hampered because of large temperature and density gradients [30]. A 19 cm long line-of-sight optical path probed both the center ∼3 cm diameter (hot) plasma core and the (cool) outer parts of the reactor, respectively. Complementary measurements were carried out with a 14 cm long absorption length to compensate for the dominant absorption contributed by the cool periphery region [56]. The model calculations confirmed the experimental results that any hydrocarbon precursor is converted into a gas mixture dominated by CH_4_ and C_2_H_2_. The equilibrium between these products is very sensitive to the gas temperature *T* and the local C/H ratio (*i.e.*, the H density). CH_4_ was found to be dominant at low C/H ratios and at *T* < 1,400 K and is prevalent in the outer part of the reactor while C_2_H_2_ is more apparent in the transition region of the reactor between hot plasma ball and the cooler parts closer to the walls of the reactor. The spatial distribution of products and the strong temperature difference (330 K up to > 2,000 K) were corroborated by comparing two CH_4_ lines of different rotationally excited levels for both absorption path lengths (*i.e.*, different interaction lengths with cool background gas) [56].

Since the MW discharge was usually operated with pressures higher than 50 mbar rapid passage effects did not occur. Potential power saturation was also not observed in preliminary experiments (see e.g.*,* figures 3a/b). Thus, a calibration of individual absorption lines was unnecessary. The temperature dependent line strength in this inhomogeneous plasma—which is of course not associated with p-QCLAS—remained the main spectroscopic concern. Although the continuous 4 cm^−1^ spectral sweep provided access to the main stable products in a spread of energy levels, the measured absorptions are biased toward species being present in the cool parts of the reactor. This is a consequence of commonly reduced line strength values in the hot plasma ball. Firstly, it transpires from these studies that relying on measured ground state densities may underestimate the product number densities (e.g., C_2_H_2_) considerably. Secondly, even if a broad spectral range is covered, which also enables excited (molecular) species to be detected, the presence of strong gradients in temperature hampers a straightforward quantification of (total) product concentrations. The absorption cross section often decreases at higher temperatures and therefore so does the sensitivity in the hot parts of the plasma. In other words, not only molecular concentrations, but also the sensitivity varies along the line-of-sight. The sum of these effects specifically favors the detection of ground state densities rather than excited states. Hence, the approach may fall short of providing useful number densities of product species.

**Figure 9.**
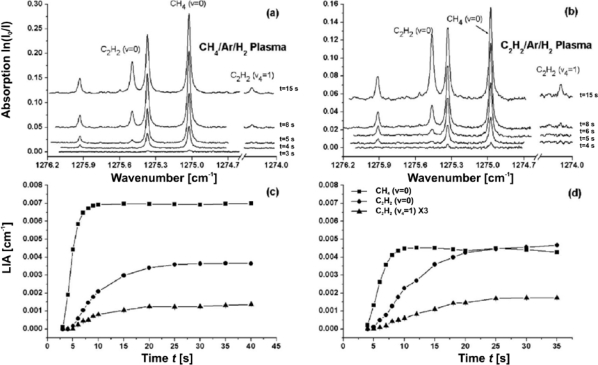
*Intra* pulse absorption spectra measured in a MW diamond deposition discharge (199.5 mbar, 1.5 kW, 565 sccm total gas flow) during addition of **(a)** 25 sccm CH_4_ and **(b)** 12 sccm C_2_H_2_ to a pre-existing 40 sccm Ar—(balanced) H_2_ discharge. Plots **(c)** and **(d)** show the LIAs for the CH_4_ (ν = 0), C_2_H_2_ (ν = 0), and C_2_H_2_(ν_4_ = 1) transitions as a function of time (reprinted with permission from [47]. Copyright 2009, American Institute of Physics.).

An experimentally interesting aspect of these studies was the time resolved determination of CH_4_ and C_2_H_2_ LIAs during the addition of either methane or acetylene as precursor to a pre-existing Ar-H_2_ discharge. The 200 μs pulse repetition rate of the QCL and binning of successive spectra (see Figure 7c) lead to a an effective time resolution of 1 s. Figure 9 depicts selected spectra obtained in such a way (Figure 9a/b) and the corresponding time traces of ground state CH_4_ as well as ground state and vibrationally excited C_2_H_2_ (Figure 9c/d). CH_4_ is apparently the dominant molecule since the absorption path covers mainly the cool part of the reactor. The flow pattern of the injected precursors from the top of the reactor towards this region and the observed low C/H ratio at early times establish the trends in Figure 9c/d.

#### 2.5.3. Diagnostics of flames

The C_x_H_y_ chemistry is also addressed in diagnostic studies of flames and combustion processes, because complex hydrocarbon precursors are present in these environments. *In-situ* chemical sensing is often affected by a combination of several of the challenges discussed above and involves

- high gas temperatures,
- weaker absorption features due to high temperatures (*i.e.,* reduced cross sections),
- large spatial inhomogeneity,
- small absorption volumes,
- scattering caused by soot formation, and
- turbulent gas flows.

It should be noted that, especially, (a) and (d) are counterproductive in terms of the sensitivity. The last factor, (f), makes p-QCLAS and specifically the long pulse mode of operation attractive for this field of gas phase spectroscopy. In contrast to other modes of operation the acquisition time for an entire spectrum in the *intra* pulse mode is below the typical time constants of environmental variations. In other words, the spectrometer analyses a “frozen” flame [66]. Additionally, the experiments are not affected by rapid passage obstacles due to sufficient collisional relaxation at atmospheric pressure.

The potential of *in-situ* p-QCLAS measurements in flames has recently been reported [66]: C_2_H_2_ as a precursor of soot formation was detected in an ethylene-air opposed flow burner. QCL pulses of 6.5 μs and a repetition period of 1 ms permitted a spectral sweep of 6.5 cm^−1^ around 1,275 cm^−1^ (7.84 μm). Since time resolved studies were not the centre of interest some thousand spectra were averaged. The relatively broad spectral coverage enabled the gas temperature to be determined from the relative intensities of H_2_O spectral lines in this range. Spatially resolved number densities of acetylene produced in the flame could be measured. Though sensitivity issues tended to be the main limiting factor further work may convert this proof-of-principle result into a useful diagnostic method for flames and exhaust plumes since it is not limited by fluctuation noise [67].

#### 2.5.4. Detection of fluorocarbons under low pressure conditions

Identification and quantification of species produced in gaseous fluorocarbon (C_x_F_y_) containing environments, e.g.*,* in plasma processes, is another field of application of high-resolution IR-LAS [68]. Specific extension to p-QCLAS has so far been reported for the transient molecule CF_3_ [57] and stable fluorocarbon molecules (CF_4_, C_3_F_8_ [69,70]). In both cases the experiments were carried out under low pressure conditions. Inherent difficulties due to non-linear absorption effects were common along with spectral overlap of absorption features in the case of C_x_F_y_ compounds thereby reducing the selectivity of the method. Complex spectra from the absorption bands of CF_4_, C_2_F_6_, C_3_F_8_, CF and CF_3_ cover the entire spectral region between 1,250 cm^−1^ and 1,300 cm^−1^ (7.7 ... 8.0 μm) which could lead to difficult multi-species analysis of experimental results. Nevertheless, employing p-QCLAS can be advantageous for studying radical kinetics to yield or refine rate coefficients since it combines high-resolution IR-LAS with a rapid spectral sweep and hence the high time resolution facility of pulsed QCLs. At first, the analysis of (low-pressure) p-QCLAS measurements of fluorocarbon species has therefore to address:

- the discrimination between blended complex spectra, and
- non-linear absorption phenomena.

Next, the time traces of the molecules of interest can be scrutinized to gain a better insight into the kinetics. Discrimination between stable reaction products (issue i) has been reported for experiments in a pulsed CF_4_/H_2_ RF plasma. A double pass configuration (∼ 90 cm absorption path) was used at 0.1 mbar total pressure. Absorption signals from spectral micro-windows in two spectral regions at 1,271 cm^−1^ and 1,274 cm^−1^ which were covered by 300 ns pulses of a QCL at two different heat sink temperatures (Figure 5a) were deconvoluted to extract CF_4_ and C_3_F_8_ number densities. The time resolution was set by the QCL repetition rate of 5 ms while spectral averaging was achieved over 25 successive plasma pulses (see Figure 7b) [69]. The retrieved number densities were based on calibrated effective absorption cross section for CF_4_ and C_3_F_8_ (issue ii). In particular for the latter species no individual absorption features could be resolved due to spectral congestion. The temperature dependence of σ_eff_ was established by complementary experiments. Since only a moderate increase in the gas temperature was expected for this kind of pulsed RF plasma the calibration of σ_eff_(*T*) was performed in a reference cell which was externally heated by a heating tape up to ∼ 380 K. A relatively pronounced change in σ_eff_ was found for CF_4_ and C_3_F_8_, e.g., for CF_4_ an increase in σ_eff_ of almost 50% at 380 K was detected [58]. Assuming an almost top-hat shaped mean gas temperature *T*(*t*) number densities were calculated for both the target (CF_4_) and product (C_3_F_8_) species. However, the temporal behavior and absolute values deduced for C_3_F_8_ did not accord with expectations for a stable higher fluorocarbon product and with complementary Fourier Transform IR measurements. It transpired that contributions from vibrationally excited molecules (e.g., CF_4_)—which are generally difficult to calibrate—have to be considered in the multi-species analysis [70].

**Figure 10.**
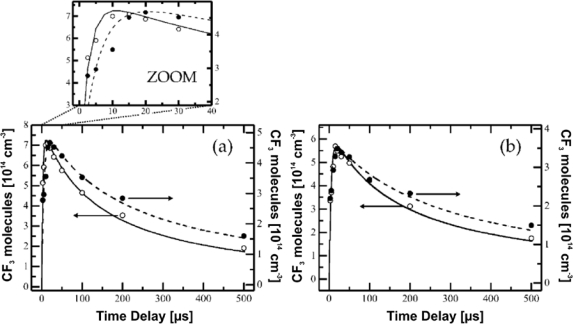
CF_3_ number densities monitored as a function of time after the CF_3_I photolysis pulse for different pump pulse energies [**(a**) 29 mJ/pulse and (**b**) 14 mJ/pulse]. Results are plotted for two pressure values: 5.32 mbar (open symbol/solid) and 2.66 mbar (full symbol/dashed), respectively (reprinted with permission from [57]. Copyright 2008. American Chemical Society).

Another study concerned the production of CF_3_ during the photolysis of CF_3_I [57]. Spectroscopic data for CF_3_ lines obtained by spectral simulation were corrected by a factor to account for all non-linear absorption phenomena in the experimental 2.7 ... 5.3 mbar pressure range used (issue ii). Similar to C_3_F_8_, almost no structure was visible in the spectrum of C_2_F_6_, which is the main stable product due to CF_3_ recombination. It was therefore treated as an effective baseline contribution (issue i). Measurements were performed in direct single pass absorption spectroscopy using a glass cell of 50 cm length. A delayed trigger configuration was applied to achieve a time resolution of 5 μs at ∼1,253 cm^−1^ (7.98 μm). The CF_3_ density was monitored as a function of time after the photolysis laser pulse (Figure 10) and rate coefficients for both the vibrational relaxation and recombination of CF_3_ were extracted. Although the typical gas temperature inhomogeneities were absent in this first *in-situ* detection of a molecular radical, another feature of plasmas namely the formation of vibrationally excited products (Section 2.5.2.) and unknown relaxation channels remained as the main source of uncertainty of the diagnostics approach. In contrast to hydrocarbon plasmas the analysis of unblended C_x_F_y_ absorption lines arising from different energy levels is hampered by the complex nature of the spectra. Nevertheless, a carefully selected spectral range encompassing minimal spectral overlap of different species makes p-QCLAS a reasonable tool for time resolved process monitoring and provides a link to industrial applications.

#### 2.5.5. Industrial etch process monitoring

Etch and deposition processes play a fundamental role in industrial plasma processing, e.g., as a part of integrated circuit processing. Increasing aspect ratios are ongoing targets in semiconductor industries and require sensitive and reliable monitoring tools and control systems for high volume production processes. The absolute densities of specifically selected precursors or etch products are desirable. Hence, QCLAS can be a powerful alternative to conventional diagnostic methods. However, in contrast to the research laboratory several specific criteria have to be addressed in industrial environments. Among them are clean room operation, real-time analysis and monitoring, *in-situ* diagnostics of the bulk plasma (rather than exhaust gas sensing) and highly non-intrusive characteristics. Additionally, synchronization and communication between the p-QCLAS spectrometer and the etch process control tool is essential in real-time monitoring of automated industrial processes.

The implementation of all these requirements has been demonstrated for *in-situ* process monitoring during industrial dynamic random-access memory fabrication. SiF_4_ was detected as a key product in a specially designed industrial dual-frequency RF etch reactor using NF_3_ as precursor [32,64]. The two channel spectrometer based on the quantum cascade laser measurement and control system (Q-MACS) [5] was equipped with a reference channel to stabilize the QCL emission by means of a C_2_H_4_ absorption line. Direct absorption spectroscopy employing only one reactor port was realized in a double pass 1.08 m long configuration while the IR radiation was guided to the reactor via a mid-IR optical fiber. Measurements were performed with an integration time of 1 s for several hundred 1 ms spectral sweeps based on the *inter* pulse method (see Figure 7d). The typical etch conditions involved a working pressure of 0.333 mbar. Non-linear absorption phenomena in the complex SiF_4_ spectrum were clearly present. Therefore, calibration of an effective absorption cross section around 1,028 cm^−1^ (9.73 μm) was used. The spectral micro-window was selected in such a way that the spectral overlap with the NF_3_ precursor in this spectral range was minimized. In contrast to CF_4_ and C_3_F_8_ (Section 2.5.4.) the temperature dependence of σ_eff_(SiF_4_) was negligible up to 380 K [32]. It should therefore be noted that σ_eff_(*T*) has to be carefully established for each molecule and spectral micro-window.

**Figure 11.**
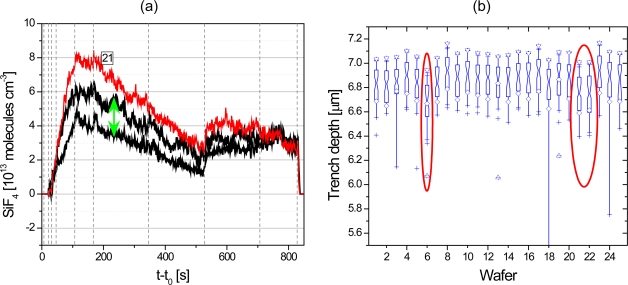
**(a)** SiF_4_ number densities as a function of time (*t* – *t*_0_) during the etch process of 25 wafers (results are shown for the last 11 runs). The arrow denotes the range of scatter (heavy solid black lines) during 11 runs. A run (21, thin solid trace) exceeding the typical scatter is plotted separately. Single-process steps are shown by vertical dashed lines. **(b)** Box plot of deep-trench etch depth of the processed wafer: the symbols represent the mean value (diamond), the first percentile (triangle up), and the 99^th^ percentile (triangle down) of the distributions. The 25th - 75th percentile range is shown by notched boxes, and their whiskers symbolize the 10th – 90th percentile range. Outliers are marked by crosses. The trench depth values are homogenously distributed around 6.9 μm except for the wafers 6, 21, and 22. The deviations (marked in red) correlate with those found in **(a)**. More details concerning individual plots and symbols can be found in [64] (figures are reprinted in part from [64] © 2009 IEEE).

Figure 11 compares results obtained by p-QCLAS (Figure 11a) during batch processing, *i.e*., deep-trench etching, of 25 wafers with inline data of the deduced trench depth of these wafers (Figure 11b) [64]. For about half of the processed wafers a common SiF_4_ number density range can be detected (Figure 11a). However, the run indicated (No. 21) was clearly not enclosed by both envelope traces which could be correlated to an incomplete previous cleaning step. The behavior of the SiF_4_ signal also agrees well with deviations found in the trench depth for this wafer (Figure 11b). Etch product monitoring in industrial environments may therefore be considered as powerful tool to detect and tackle process errors in real-time.

The communication between the spectrometer and the etch chamber control tool was required in this case for an intermediate baseline acquisition to facilitate corrections due to the long-term degradation of the transmission properties of the optical components [64]. Furthermore, the QCL spectrometer was not only designed for gas phase absorption spectroscopy parallel to the processed wafers, but also for an interferometer arrangement perpendicular to the etched surface. Using this configuration SiF_4_ absorption in the gas phase was almost negligible due to the short absorption path length. The detected signal allows then etch rate determination and end point detection. Changes in the refractive index and material thickness cause phase shifts and changes in frequency and amplitude of the observed IR interferences. This provides an alternative and chemically selective means of end point detection compared with conventional optical emission spectroscopy or mass spectrometry.

#### 2.5.6. Industrial process control

**Figure 12.**
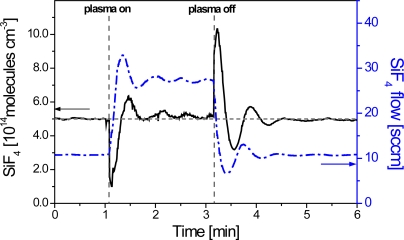
Stabilization of the SiF_4_ density (solid) around the target value of 5 × 10^14^ cm^−3^ (dotted trace) in a SiF_4_-N_2_ MW plasma (0.2 mbar) by actively controlling the feed gas flow (dash-dotted trace). (reprinted with permission from EDP Sciences (http://www.epjap.org/) [33]).

In the previous section (passive) synchronization between an IR-LAS spectrometer and a plasma process control tool was discussed. The p-QCLAS spectrometer has recently been adapted to achieve active control of a discharge by means of a feedback loop based on *in-situ* measurements employing a two laser arrangement [33]. Two pulsed QCLs emitting at 1,028 cm^−1^ and 973 cm^−1^ were used in the *inter* pulse mode to measure SiF_4_ and C_4_F_6_, respectively, in a MW reactor. A feedback signal was derived from the acquired molecular concentrations which served to stabilize the amount of feed gas at a target value by adjusting the corresponding mass flow controllers. This application was demonstrated for a SiF_4_-C_4_F_6_-N_2_ gas mixture at 0.3 mbar total pressure [33]. Adjusting the SiF_4_ feed gas flow (at 0.2 mbar total pressure) was also demonstrated in a SiF_4_-N_2_ MW plasma as illustrated in Figure 12. Precursor fragmentation and the subsequent flow control changes causes the SiF_4_ density to fall below or exceed the target value at the plasma off-on transitions. Stabilization was achieved after ∼ 1 min which is strongly dependent on the reactor geometry and the applied gas flow rates.

## 3. Application of CW-QCLs

DFB–cw–QCLs are increasingly available and combine the advantages of pulsed lasers, such as near room temperature operation and continuously tuneable single mode emission, with straightforward and convenient tuning options transferred from TDL absorption spectroscopy (TDLAS), e.g., sophisticated sweep integration systems [71]. The application of cw–QCLs enables a higher *SNR* to be achieved due to the higher output power levels of QCLs compared with TDLs. Additionally, intensity fluctuation which are inherent for pulsed QCLs are considerably reduced. Due to the constant base current fed to the cw laser a frequency chirp is absent and hence rapid passage effects cannot occur at low pressure.

The application of cw–QCLAS for plasma diagnostic purposes is beginning to appear (3.1.) and will clearly be advantageous. First examples concerning precursor monitoring in industrial (3.1.1.) and research (3.1.2.) environments or the potential combination with high finesse optical cavities (3.2.), which was difficult to achieve with pulsed QCLs, are discussed below.

### 3.1. Direct Absorption Employing cw-QCLAS

#### 3.1.1. Deposition rate and precursor dissociation studies in silane plasmas

An early application of cw-QCLAS for silane detection has been reported. A home-built cw-QCL spectrometer was applied to an industrial PE-CVD reactor used for silicon thin film deposition. The TE cooled DFB-laser was tuned between 2,242 cm^−1^ and 2,244 cm^−1^ (∼ 4.27 μm) to record the R(9) multiplet of SiH_4_. The silane absorbance in the 3.7 m long exhaust line of the reactor was monitored during the low pressure (< 5 mbar) deposition process. The deposition rate of microcrystalline silicon films was estimated from the depletion of the precursor concentration in SiH_4_ plasmas diluted with H_2_ and agreed well with complementary *ex-situ* profilometric measurements [72]. Since this experiment was not designed for retrieving absolute silane number densities the precursor depletion could be inferred from the ratio of the SiH_4_ absorbance measured when the plasma was switched on and off, respectively. Hence, high resolution spectroscopic data were not required. The experiments were carried out by employing direct absorption spectroscopy in single pass configuration and achieved a sufficient *SNR*. It can therefore be concluded that spectrometers based on cw-QCLAS are an attractive new option for industrial process analysis.

#### 3.1.2. CH_4_ detection in a microwave discharge

**Figure 13.**
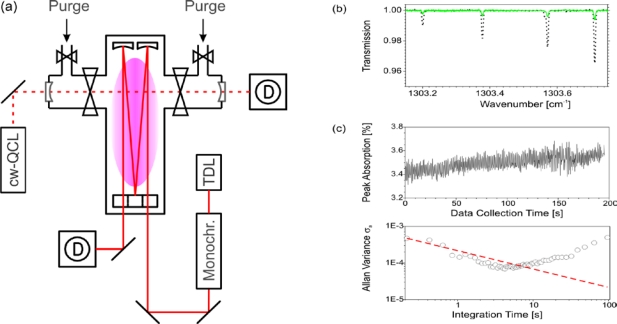
**(a)** Schematic diagram of the experimental setup used for IR-LAS with a planar MW discharge reactor. A 7.67 μm cw-QCL was guided through purged viewing apertures perpendicular to a lead salt TDL spectrometer aligned to achieve 24 passes within an *in-situ* White cell having a 1.5 m mirror separation (*L*_TDL_ = 36 m). The single pass QCL absorption path (reactor clearance) was *L*_QCL_ = 0.2 m. **(b)** CH_4_ transmission spectra measured with the cw-QCL in a CH_4_-Ar plasma [25 sccm + (225 sccm + 20 sccm purge gas), 1.5 mbar, 0.7 kW input power, dotted line] and an O_2_-Ar plasma [20 sccm + (15 sccm + 25 sccm purge gas), 1.5 mbar, 0.7 kW input power, solid line]. **(c)** Upper: CH_4_ peak absorption at 1,303.71 cm^−1^ as a function of time in the CH_4_-Ar discharge shown in (b). Lower: Allan plot retrieved from the data stream plotted in the upper panel (symbols). The trend in white noise is indicated (dashed line).

Another spectrometer was combined with a planar MW discharge reactor to quantify the sensitivity available from cw-QCLAS. The vacuum vessel was specifically designed for high resolution IR-LAS studies on (transient) molecular species and therefore equipped with a White type multiple pass cell of 1.50 m mirror separation. A relatively homogeneous plasma can usually be achieved across the entire absorption volume. More details about the plasma reactor are available in [73,74]. The QCL spectrometer was mounted perpendicular to the long axis of the reactor to measure the precursor concentration parallel to a cross sectional plane of 0.20 m length direct single pass absorption configuration. Figure 13a shows a schematic diagram of the experimental arrangement. The reactor was also equipped with a lead salt TDLAS instrument which was aligned in such a way that 24 passes were achieved with the White cell optics. Details of this IR Multi-component Acquisition system (IRMA) can be found elsewhere [75]. The TDL spectrometer provided complementary measurements around 1,385 cm^−1^ which were used to validate the cw-QCLAS results.

The TE cooled cw-QCL was swept in frequency by impressing a 1 ms ramp on the operating current, known as the sweep integration method used in TDLAS [71], to achieve a 0.8 cm^−1^ spectral scan. The IR radiation centered around 1,304.5 cm^−1^ (7.67 μm) was detected with a standard liquid nitrogen cooled HgCdTe detector. The reactor viewing apertures were purged with an Ar gas flow (Figure 13a). Argon was selected as purge gas here, since it is usually the main precursor in the gas mixtures of the MW discharges. Since the purge gas flow influences the effective QCLAS absorption path *L*_QCL_ its flow rate had to be established first. While adjusting the Ar purge flow the CH_4_ mixing ratios of the gas in the reactor obtained by the TDL (*L*_TDL_ = 36 m = const.) and the QCL spectrometer, respectively, were compared. About 20 sccm were sufficient to prevent the IR optics from being degraded and to yield *L*_QCL_ = 0.20 m. Thus, *L*_QCL_ matches the distance between the reactor walls.

Examples of cw-QCLAS transmission spectra for CH_4_ measured in an Ar-(9 %) CH_4_ and an Ar-(33 %) O_2_ plasma are depicted in Figure 13b. Using the multiple pass cell optics in combination with TDLAS an (effective) gas temperature in the range of (600 ... 700) K along the line-of-sight was normally determined. However, complementary measurements, detailed in the following Section 3.2., highlighted similar temperature and density gradients to those pointed out earlier: significant inhomogeneities have been demonstrated for the diamond deposition reactor in Section 2.5.2. The effective absorption path lengths in this PE-CVD vessel and over the 0.20 m cross sectional view in our MW reactor are of the same order. Hence, number densities were not extracted from the present cw-QCLAS measurements. Nevertheless, the results can qualitatively be discussed. The absorption of the strongest CH_4_ line covered by the spectral scan was lowered from 57% without plasma to some 3% (Figure 13b) when the discharge was on. This suggests efficient precursor dissociation. The *SNR* was also good enough to measure CH_4_ formed in an Ar-O_2_ discharge. A CH_4_ peak absorption of ∼ 0.5% was detected at 1,303.712 cm^−1^ though methane was not added to the discharge when the plasma was ignited. The CH_4_ signal steadily decreased over tens of minutes. This suggests etching processes of amorphous carbon containing layers covering the reactor walls. Both the effects of efficient CH_4_ depletion and etching processes have already been reported for this reactor [76].

The peak absorption of the strongest CH_4_ feature was followed with time to determine the sensitivity of the spectrometer by means of the Allan variance σ_a_ (Figure 13c) [77,78]. An integration time of 0.2 s corresponding to a spectral average of 200 individual sweeps (see Figure 7d) was used. The peak absorption sensitivity was found to be 2 × 10^−4^ (4 × 10^−4^) for a reasonable integration time in plasma diagnostics of 1 s (0.2 s). The Allan minimum of 0.7 × 10^−4^ was found to occur at 4 s integration time with plasma on and at 15 s with the discharge off (the latter not shown in Figure 13c). The main limiting factor may be associated with drifts in the precursor density caused by the plasma rather than weak drifts in the unstabilized QCL frequency as would be the case for measurements without plasma. The presently established sensitivity (10^−3^ cm^−1^Hz^−1/2^) corresponds to a CH_4_ number density for the present conditions (1.5 mbar, 0.2 m absorption path, and assumed 296 K, due to the unknown gas temperature in the plasma) of 7 × 10^11^ cm^−3^ or 19 ppm. This limit of detection was achieved in the single pass configuration without reducing the noise by excessive spectral averaging, post-processing of data or applying modulation techniques.

### 3.2. Chemical Sensing in Plasmas Using Optical Cavities

Increasing the sensitivity of (IR) plasma diagnostic methods is desirable either for lowering the limit of detection to levels where important transient species might be expected to be produced in the plasma or for obtaining better spatial resolution, *i.e.,* for reducing the line-of-sight to obtain more precise local molecular number densities. Both aims may be contradictory, because increased effective absorption path lengths *L*_eff_ are required in the former. In conventional linear absorption spectroscopy these are often realized by folding the laser beam in multiple pass optics. However the combination of (*in-situ*) long path length optics with standard reactors is difficult and may require specifically designed plasma vessels, e.g., as demonstrated in the previous section. An alternative approach for stable species is *ex-situ* sampling in multiple pass cells which may provide up to 200 m absorption length and sensitivities of 3 × 10^−10^ cm^−1^Hz^−1/2^ at the expense of large sampling volumes [79]. Increasing the effective absorption length by employing high finesse optical cavities [80,81] can provide both increased sensitivities at inherently small base lengths (e.g., [34,40,82] and references therein) and would therefore be ideally suited to localized measurements in discharge environments. The application of cw-QCLs to CEAS methods benefits from the milliwatt output power without being hindered by the frequency chirp as reported for pulsed QCLs [40]. Without any rapid spectral chirp present the effective line width is now only limited by the performance of the power supply and is typically of the order of ∼ 0.001 cm^−1^ (30 MHz) [79,83,84]. The number of excited cavity modes is considerably reduced and the intensity gain in the developing modes is not limited by the (fast) sweep rate α. Furthermore, CEAS is relatively straightforward to implement if sophisticated cavity stabilization schemes or laser locking by optical feedback is omitted.

Basically, the cw-QCLAS experiments detailed in Section 3.1.2. served to prepare for CEAS measurements on a MW plasma. For this purpose the KBr windows of the viewing apertures were replaced by high reflectivity mirrors (Figure 13a). The laser setup and optical arrangement was essentially the same as described in [40]. Now the QCL radiation was guided through the reactor (again perpendicular to the White cell optics). Since the purge gas flow for the mirrors was already optimized, the theoretical effective absorption path *L*_eff_ follows from the base length (0.2 m) multiplied by the enhancement factor (1 – *R*)^−1^, where *R* is the calibrated mirror reflectivity. In what follows two different cw-QCLs are employed to measure HCN around 1,304 cm^−1^ (Section 3.2.1.) and to detect NO at 1,819 cm^−1^ (5.50 μm) (Section 3.2.2.).

#### 3.2.1. HCN detection in an Ar-CH_4_-N_2_ microwave plasma

The cw laser used for HCN detection was the same as for the direct absorption experiments, but slightly shifted in frequency. The spectral scan rate had to be adjusted to 1.2 ms per 0.5 cm^−1^ sweep to permit a better intensity build-up on the cavity modes which were separated by ∼ 0.006 cm^−1^ (*i.e.,* 80 cm mirror separation). The effective reflectivity of the mirrors was determined to be *R* = 99.965 % which transferred to *L*_eff_ would yield almost 600 m along the cross sectional reactor plane. HCN was simultaneously detected by means of the lead salt laser based IRMA equipment which was tuned to 1,396.78 cm^−1^ to detect the *P*5 line (*S*(296 K) = 3.9 × 10^−20^ cm/molecule [85]).

**Figure 14.**
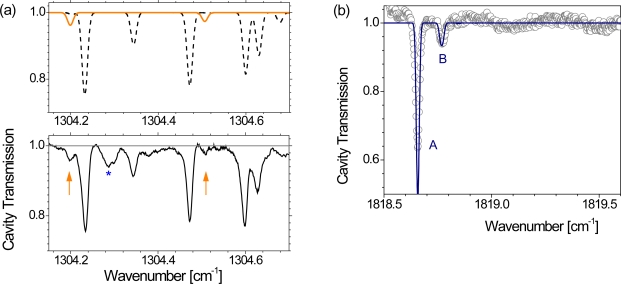
**(a)** Transmission spectrum obtained by cw-QCL-CEAS spectrometer mounted on the MW reactor in Figure 13a. Molecular absorption lines were detected *in-situ* in a 395(+ 25)/10/10 sccm Ar(+ purge)/CH_4_/N_2_ plasma (1.5 mbar total pressure, 1.5 kW): The HCN lines, *P*34*e* and *P*38, are indicated by arrows. Potential H_2_O absorption is marked with *. Strong CH_4_ lines would require a correction (not within the weak absorption limit). Upper: Calculation of a transmission spectrum for CH_4_ (400 K, dotted trace) and HCN (1200 K, solid trace). Since the gas temperature (gradient) and thus number density (gradients) are unknown this plot should be regarded as a guide to the eye for identifying the main features in the lower panel. **(b)** CEAS spectrum of NO (experimental - symbols) measured in an Ar-O_2_ MW plasma [15(+ 25)/20 sccm Ar(+ purge)/O_2_, 0.5 mbar total pressure, 1.5 kW]. The calculated spectrum (solid trace) again serves only as a guide to the eye and reasonable agreement was only found by assuming clearly different gas temperatures for features A and B. Experimental (peak) absorption of A requires a correction.

The measurements were performed in an Ar-CH_4_-N_2_ plasma using the operating conditions (1.5 kW input power, 1.5 mbar total pressure, 395(+ 25)/10/10 sccm Ar(+ purge)/CH_4_/N_2_) to provide a link to earlier studies [76]. The HCN mixing ratios measured by means of the TDL spectrometer were essentially of the same order (0.4%) assuming an effective gas temperature of about 600 K along the line-of-sight (*L*_TDL_ = 36 m). A cavity transmission spectrum showing mainly HCN and CH_4_ features is presented in Figure 14a (lower panel). Due to efficient precursor depletion the CH_4_ lines are sufficiently decreased not to interfere with the *P*34*e* line of HCN at 1,304.20 cm^−1^ while an overlap of the *P*38 line at 1,304.51 cm^−1^ with CH_4_ could still be detected.

Two aspects of the CEAS spectrum in Figure 14a should be highlighted. Firstly, the (peak) ratio of the CH_4_ lines - though already violating the weak absorption assumption and usually requiring a non-linear correction for quantitative spectroscopy - suggests a gas temperature of only ∼ 400 K. Assuming that the viewing apertures are purged, this result is surprising, since most of the 0.2 m cavity base length, *i.e.,* the reactor clearance, was supposed to be the active or hot part of the MW plasma.

A reasonable explanation might be given in the work of Ma *et al.* (Section 2.5.2., [56]): the observed CH_4_ is detected and formed only in the cool outer parts of the line-of-sight. On the other hand, the HCN lines in the spectral range covered by the cw-QCL are too weak at low temperatures (*S_P_*_34_*_e_*(296 K) = 3.9 × 10^−24^ cm/molecule [85]), *i.e.*, 10^4^ times weaker than the *P*5 line measured by the IRMA system (due to a 55 times higher lower state energy [85]). However, HCN was still detected, although the gain in *L*_eff_ by the CEAS spectrometer cannot be higher than 15 compared with *L*_TDL_. Hence, the HCN produced in the plasma might be present in different rotationally and vibrationally excited states (similar to C_2_H_2_ in [56]) and a unique HCN number density cannot easily be extracted without detailed chemical modeling. The calculated transmission spectrum in Figure 14a (upper panel) should therefore only be considered as a guide for the eye. The absorption feature around 1,304.29 cm^−1^ could not be assigned, but is potentially caused by H_2_O, although O_2_ was not fed to the reactor during the measurements.

#### 3.2.2. NO detection in an Ar-O_2_ plasma

The cw-QCL employed for the Ar-CH_4_-N_2_ plasma experiment described above was replaced by a second laser tuned to detect NO around 1,818.65 cm^−1^. The cavity mirrors had to be used at the edge of their high reflectance regime. An effective reflectivity of *R* = 99.82 % was established by a rudimentary calibration procedure. The upper limit *L*_eff_ for the reactor would therefore be ∼ 115 m. The experimental CEAS spectrum of an Ar-O_2_ plasma (Figure 14b) clearly exhibits two groups of unresolved NO features at 1,818.66 cm^−1^ and 1,818.78 cm^−1^, respectively. The unexpected observation of NO in an Ar-O_2_ discharge was probably caused by a tiny leak in the sealing of the quartz windows used for coupling the MW radiation into the reactor volume.

A straightforward analysis of CEAS spectra employing the theoretical *L*_eff_ and thus a quantification of the NO density was again hampered by potential inhomogeneities along the line-of-sight though it is only 0.2 m. The detection of both NO features (A and B in Figure 14b) suggests a strong gradient in *T*, since the lower state energy *E*'' of both groups of unresolved lines differs significantly. The *E*'' value of transitions which are detected as A is 557.8 cm^−1^ whereas it is 2,008.5 cm^−1^ for B (energy values in *E*/(hc), where c is the velocity of light [85]). In other words, a fit to the experimental results assuming a single gas temperature was not feasible, as pointed out for the CEAS spectrum containing CH_4_ and HCN in Figure 14a. The line of weaker absorption (B) required *T* > 1,000 K be detectable. The strong line (A) exceeds the weak absorption assumption and would usually need a correction for quantification. Nevertheless, assuming a reasonable upper limit NO mixing ratio of a few percent the fit to the (uncorrected) strong absorption feature (A) can only be achieved for gas temperatures below 700 K. A correction factor would increase the detected absorption which requires even lower temperatures in the calculations. The CEAS approach thus provides sufficiently long effective absorption path lengths to measure and identify molecular species in different energy levels while the detection volume remains small.

## 4. Conclusions

QCLs have been available for more than a decade and in the interim have been extensively used for highly sensitive trace gas sensing spectrometers. However, their specific application for plasma diagnostic purposes has only recently been recognized. Several QCLAS studies in non-thermal plasma environments have been reported in the literature so far and are collected in Table 2. Selected examples, among them investigations of atmospheric and low pressure plasmas, have been discussed focusing particularly on time resolved measurements and kinetic studies. This overview was also concerned in part with non-linear absorption phenomena (e.g., the rapid passage effect) associated with pulsed QCLs under low pressure conditions. Another focus was on industrial applications, particularly of p-QCLAS. Real-time analysis now enables plasma processes to be controlled by employing feedback loops, e.g., to mass flow controllers.

The significance of a detailed knowledge of the gas temperatures in discharges has been highlighted as the line strength, for example, is very sensitive to even small changes in temperature. Hence the increased temperatures accompanied by inhomogeneities across the plasma volume, often absent in trace gas sensing spectrometers, have to be carefully considered. We also highlighted the fact that intermediate molecules formed in the plasma are often present in different (vibrationally) excited levels. Detecting a specific transition, e.g., of a lower state energy, may therefore significantly underestimate the corresponding number densities and provide insufficient input data for plasma chemical models.

**Figure 15.**
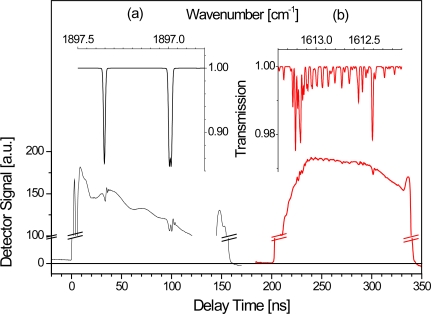
Lower: Experimental spectra of two multiplexed pulsed QCLs synchronized to a trigger event at *t* = 0. Upper: Calculated spectra for the specific emission ranges of both QCLs. **(a)** Rapid spectral sweep at ∼1,897 cm^−1^ (5.27 μm) during a 150 ns QCL pulse showing absorption features of NO (50 cm absorption length, 2.66 mbar). **(b)** Spectral sweep of a pulsed QCL (150 ns pulse length) emitting around 1,613 cm^−1^ (6.20 μm) to observe NO_2_ absorption lines using the same conditions as in **(a)** [65].

Another aspect of plasma diagnostics is the detection of multiple species which is challenging due to the limited spectral coverage of DFB-QCLs. A combination of QCLs using beam splitters has been demonstrated [64] and was extended to high time resolution studies by multiplexing and synchronizing the QCL pulses with the plasma (Figure 15, [65]). A promising alternative approach might be the application of external cavity lasers covering 50 ... 100 cm^−1^ which have been recently developed and employed specifically for measuring explosives [53,87–89] Further progress in QCL fabrication and performance is forthcoming and will clearly enhance the trend towards employing cw-QCLs in mid-IR based chemical sensors [90]. In contrast to pulsed lasers the rapid passage effect at low pressure conditions is absent and the stability in frequency and intensity yields an increased *SNR*. However, pulsed QCLs with their inherent rapid frequency-down chirp are still essential for highly time resolved studies where a time resolution better than milliseconds is required.

The increasingly good performance particularly of cw-QCLAS and the absence of a rapid frequency chirp with these devices facilitates the extension of CEAS methods to the mid-IR spectral range. The straightforward implementation of such detection schemes is often limited in sensitivity by the residual mode noise. In other words, multiple pass cw-QCL spectrometers may achieve a better ultimate sensitivity. However, the CEAS approach with similar effective absorption lengths offers the advantage of reduced absorption volumes and the potential of *in-situ* diagnostics. This is of special interest for *in-situ* detection of plasma produced molecular species, specifically transient molecules, which cannot be extracted to (*ex-situ*) high sensitivity trace gas spectrometers. It may also provide a better spatial resolution along the line-of-sight and thus local densities compared to *in-situ* multi pass optics. Such an improvement could be demonstrated for the first time by combining a cw-QCL with the CEAS approach to detect HCN and NO produced in a MW plasma.

An alternative approach for facilitating a better spatial resolution might be *in-situ* pump probe experiments. Although saturated absorption spectroscopy has so far mainly been applied in the field of frequency metrology [91,92] as a Doppler-free method, it could be used to obtain localized concentration information from plasmas. It has been shown [93] and partly discussed in this paper that the output power levels of QCLs are sufficiently high to obtain power saturation. Lamb-dip signals may thus provide number densities from a well-defined plasma (detection) volume in a configuration similar to well-known (two photon absorption) laser induced fluorescence techniques.

The present results obtained with a high finesse optical cavity immediately revealed strong gradients in the gas phase which usually remain undetected by applying conventional techniques. On the other hand, such inhomogeneities in the plasma require a thorough chemical modeling to extract absolute densities and gain insight into the kinetics.

## Figures and Tables

**Table 1.** Summary of potential non-linear absorption effects that might be observed with pulsed QCLs.

**Important Parameters**		
Chirp (Sweep) Rate	*α*	
Relaxation Rate	*γ*	
Transition Dipole Moment	*μ*	
Electric Field Amplitude	*E*_0_	
Normalized Sweep Rate	*A* = *α/γ*^2^	
Saturation Parameter	*Σ* ∼ *μE*_0_*/γ*^2^	

**Criterion**	**Effect and Conditions**	

***A* ≫ 1**	*Rapid Passage (Swept Gain)*	
		• high chirp rate (*α*)
		• slow relaxation (*γ*) (e.g., low pressure)
***Σ* ≫ 1**	*Power Saturation (Optical Pumping)*	
		• strong optical transition (high *μ*)
		• strong driving electromagnetic (EM) field (*E*_0_)
		• slow relaxation (*γ*) (e.g., low pressure)
***A/Σ* ≪ 1**	*Adiabatic Rapid Passage*	
		• rapid passage (frequency chirp)
		• strong optical pumping
		⇒ long interaction time between EM field and transition
***A/Σ* ≫ 1**	*Linear Rapid Passage*	
		• strong rapid passage (frequency chirp)
		• optical pumping
		⇒ short interaction time between EM field and transition

**Table 2.** Tabulation of plasma diagnostic applications of mid-IR QCLs.

**Species**	**Spectral Range [cm^−1^]**	**Type of Plasma**	**Application^1^**	**Pressue [mbar]^2^**	**Type of QCL**	**Tuning Method**	**Method of Absorpt.^3^**	**Time Resolution^4^**	**Year**	**Ref.**
CH_4_/C_2_H_2_	∼ 1275	MW	Res.	> 50 (199.5)	pulsed	intra	DAS/SP	1 s	2006 2009	[30] [56]
CH_4_	1253	RF	Res.	0.23	pulsed	intra	DAS/SP	n.a.	2008	[55]
C_2_H_2_	∼ 1275	Flame	Res.	1013	pulsed	intra	DAS/SP	n.a.	2009	[66]
NO	1897	DC	Res.	2.7	pulsed	intra	DAS/SP	5 μs	2007	[31]
NO/NO_2_	1897 1613	RF	Res.	2.7	pulsed pulsed	intra	DAS/SP	200 μs	2009	[65]
CF_4_/C_3_F_8_	1271/1274	RF	Res.	0.1	pulsed	intra	DAS/DP	5 ms	2009	[58, 69, 70]
CF_3_	1253	Photolysis	Res.	2.6 ... 5.4	pulsed	intra	DAS/SP	5 μs	2008	[57]
SiF_4_ (NF_3_)	1028	RF	Ind.	0.33	pulsed	inter	DAS/DP	∼ 1 s	2007 2009	[32] [64]
SiF_4_/C_4_F_6_	1028 973	MW	Res. (Ind.)	0.2 ... 0.3	pulsed pulsed	inter	DAS/DP	1 s	2010	[33]
BCl_3_	964	MW (DC)	Ind. (Res.)	2	pulsed	inter	DAS/DP	3 s	2009 2010	[63] [86]
SiH_4_	2244	^5^VHF	Res. (Ind.)	3.5 ... 4.5	cw		DAS/SP	n.a.	2009	[72]
CH_4_	1303	MW	Res.	1.5	cw		DAS/SP	0.2	2010	*This work*
HCN	1304	MW	Res.	1.5	cw		CEAS	> 1 s	2010	*This work*
NO	1819	MW	Res.	1.5	cw		CEAS	> 1 s	2010	*This work*

1 - Res. = research environment, Ind. = industrial application

2 - converted into mbar, if necessary

3 - D-AS = direct absorption spectroscopy, SP = single pass, DP = double pass

4 - n.a. = not available

5 - VHF = very high frequency

## A. Appendix

### A.1. Acronyms

AS: absorption spectroscopy
CEAS: cavity enhanced absorption spectroscopy
CRDS: cavity ring-down spectroscopy
CVD: chemical vapor deposition
cw: continuous wave
D-AS: direct absorption spectroscopy
DC: direct current (discharge)
DP: double pass
EM: electromagnetic
FM: frequency modulation
DFB: distributed feedback
IR: infrared
ICL: inter-band cascade laser
ICOS: integrated cavity output spectroscopy
LAS: laser absorption spectroscopy
LIA: line integrated absorption coefficient
MW: microwave (discharge)
NICE-OHMS: noise immune cavity enhanced optical heterodyne molecular spectroscopy
OA: off-axis
OF: optical feedback
PE: plasma enhanced
QCL: quantum cascade laser
QCLAS: quantum cascade laser absorption spectroscopy
QE: quartz enhanced
cw-QCLAS: quantum cascade laser absorption spectroscopy using cw devices
p-QCLAS: quantum cascade laser absorption spectroscopy using pulsed devices
PAS: photo-acoustic spectroscopy
PS: phase shift
ppm: part per million
RF: radiofrequency (discharge)
sccm: standard cubic centimeters per minute
SP: single pass
*SNR*: signal-to-noise ratio
TDL: tuneable diode laser
TDLAS: tuneable diode laser absorption spectroscopy
TE: thermoelectric
VHF: very high frequency (discharge)
WM: wavelength modulation

### A.2. Chemical formulae

Ar: Argon
BCl_3_: Boron trichloride
CF: Fluoromethylidyne
CF_3_: Trifluoromethyl
CF_4_: Carbon fluoride (Tetrafluoromethane)
C_2_F_6_: Perfluoroethane
C_3_F_8_: Perfluoropropane
C_4_F_6_: Hexafluorocyclobutene
CF_3_I: Trifluoroiodomethane
CH_4_: Methane
C_2_H_4_: Ethylene
C_2_H_2_: Acetylene
H_2_: Hydrogen
HCN: Hydrogen cyanide
H_2_O: Water
N_2_: Nitrogen
NO: Nitric oxide
NO_2_: Nitrogen dioxide
O_2_: Oxygen
NF_3_: Nitrogen trifluoride
SiF_4_: Silicon tetrafluoride
SiH_4_: Silane
